# Developments of Recent Applications for Early Diagnosis of Diseases Using Electronic-Nose and Other VOC-Detection Devices

**DOI:** 10.3390/s23187885

**Published:** 2023-09-14

**Authors:** Alphus Dan Wilson

**Affiliations:** Pathology Department, Center for Forest Health & Disturbance, Forest Genetics and Ecosystems Biology, Southern Research Station, USDA Forest Service, Stoneville, MS 38776, USA; alphus.wilson@usda.gov; Tel.: +1-662-336-4809

**Keywords:** early disease detection, clinical pathology, electronic nose, disease diagnostics, point-of-care testing (POCT), chemical sensors, disease biomarkers, metabolomics, volatile organic compounds (VOCs), white-nose syndrome (WNS)

## Abstract

This Editorial provides summaries and an overview of research and review articles published in the *Sensors* journal, volumes 21 (2021), 22 (2022), and 23 (2023), within the biomedical Special Issue “Portable Electronic-Nose Devices for Noninvasive Early Disease Detection”, which focused on recent sensors, biosensors, and clinical instruments developed for noninvasive early detection and diagnosis of human and animal diseases. The ten articles published in this Special Issue provide new information associated with recent electronic-nose (e-nose) and related volatile organic compound (VOC)-detection technologies developed to improve the effectiveness and efficiency of diagnostic methodologies for early disease detection prior to symptom development. For review purposes, the summarized articles were placed into three broad groupings or topic areas, including veterinary-wildlife pathology, human clinical pathology, and the detection of dietary effects on VOC emissions. These specified categories were used to define sectional headings devoted to related research studies with a commonality based on a particular disease being investigated or type of analytical instrument used in analyses.

## 1. Introduction

The *Sensors* Special Issue “Portable Electronic-Nose Devices for Noninvasive Early Disease Detection” was conceived and initiated to celebrate over 30 years of electronic sensor devices and technologies developed for disease detection in the field of clinical pathology since electronic-nose (e-nose) devices were first introduced in the mid-1980s. The current Special Issue is the second in a series organized and devoted to revealing new emerging electronic devices, including e-nose and related volatile organic compound (VOC)-detection technologies utilized in early disease diagnoses. The first Special Issue in this biomedical series, “Noninvasive Early Disease Diagnosis”, included six research articles published in the MDPI journal *Biosensors*, as summarized in a previous related editorial [1]. A wide diversity of sensor–system applications continue to be developed and applied to biomedical, clinical, and diagnostic (disease detection) applications. Among the key advantages of electronic instruments, designed to detect complex mixtures of VOC analytes in gaseous clinical samples, are the capabilities of achieving noninvasive early disease detection prior to symptom development. The capability of classifying and characterizing gaseous organic chemical samples into relatively simple sensory outputs makes e-noses and related electronic devices unique among diagnostic analytical instruments. These devices have the improved characteristics of simple operation, relatively low-cost, good precision, and real-time operation with high sample throughput capabilities. The research articles reviewed here from this Special Issue include studies devoted to the common goal of developing improved and effective diagnostic and analytical applications using various types of specialized electronic technologies which take advantage of changes in host VOC emissions resulting from physiological changes, dysregulations of host metabolic pathways associated with pathogenesis (during early disease development), and from changes in diet.

## 2. Review of Special Issue Contributions

This Special Issue, “*Portable Electronic-Nose Devices for Noninvasive Early Disease Detection*”, published in the MDPI journal *Sensors*, is composed of ten research articles, presenting the results of original studies conducted by research teams operating in or associated with six separate countries, including the Netherlands, the United Kingdom, Russia, Italy, Spain, and the United States. The articles summarized here represent works that involve the testing of electronic instruments and associated methodologies being developed for noninvasive early detection and diagnosis of human and veterinary-wildlife diseases, and for determining some human dietary effects on intestinal and urinary VOC emissions. The clinical sample types collected and used in these diagnostic and physiological studies, as host-derived sources for collecting headspace VOC emissions for analysis, primarily involved whole-body and breath-air samples from diseased animals such as from small bats, exhaled breath condensate and tracheal washes (from domesticated calves) and breath, urine, and colon (fecal) gas samples from human patients.

The research topic areas investigated in this Special Issue were used to categorize and group the summaries and descriptions of studies into broad groupings, including veterinary-wildlife pathology, human clinical pathology, and the detection of dietary effects on VOC emissions. These specified categories were used to define the following sectional headings devoted to related research studies that have commonalities based on a particular disease being investigated, a certain type of electronic device being used for VOC analysis or diagnoses, or specific uses of portable e-nose or related analytical devices being developed for human or animal health applications.

### 2.1. Veterinary-Wildlife Pathology

An analysis of whole-body VOC emissions from insectivorous bats using e-nose devices was recently carried out for early detection of white-nose syndrome (WNS) in bats. This necrotrophic disease, caused by the keratinophilic fungal pathogen *Pseudogymnoascus destructans* (Pd), has occurred primarily in cave-dwelling Nearctic bats in which the disease has caused a reduction in populations of at least twelve small bat species in some eastern regions of North America (up to 99%) since 2006. The research by Doty et al. [2] resulted in the development of a customized apparatus, consisting of a bat VOC air-collection apparatus assembly with a glass bat air-sampling chamber connected in tandem with a Xitech vacuum chamber, to collect whole-body VOC emissions from tricolored bats, *Perimyotis subflavus*. VOC emissions were collected within a multilayered, polyethylene-aluminum foil (PE-AL) VOC air-sampling bag inside of the vacuum chamber. Tricolored bats from three separate sampling groups, based on environmental conditions of the bat habitat, levels of physical activity, and WNS-disease status, were captured temporarily for collection of VOC missions to analyze the chemical composition of air samples as influenced by these combinations of factors and associated physiological states. VOC analysis was conducted using a dual-technology Heracles II GC/E-nose system with two flame ionization detectors (FID) for gas chromatographic (GC) analysis and a very large metal oxide semiconducting (MOS) sensor array for collecting smellprint signature data from bat air samples. This work was a follow-up study to a previous related WNS study that utilized a portable Cyranose 320 (C-320) e-nose with a carbon black polymer composite (CBPC) 32-sensor array to discriminate between nine insectivorous bat species based on differences in whole-body VOC emissions and associated smellprint signatures (VOC profiles) [3].

Gas chromatographic data showed distinctly different VOC profiles from comparisons of chromatograms of VOC emissions derived from healthy active extracave (field) bats, healthy inactive intracave bats, and WNS-diseased intracave tricolored bats [2]. The abundance and diversity of whole-body VOCs emissions decreased in magnitude (from higher to lower) in healthy field bats, torpid healthy cave bats, and torpid WNS-diseased cave bats. In addition, agricultural pesticides, primarily consisting of insecticides and herbicides from extra-cave sources, were found among minor peak area volatile emissions from some bats of all three sample types, but highest levels of pesticides occurred in healthy, active field bats (outside of caves), which presumably have a greater exposure to pesticides in the environment.

A comparative analysis of differences in chemical composition of VOC emissions from bats of each sample type resulted in the discovery of specific VOC metabolites as chemical biomarkers potentially useful as indicators of bat physiological states and/or metabolic conditions related to physical activity, consciousness, environmental location, and health status [2]. A total of thirteen VOC biomarkers were identified, including six low-to-moderate-molecular-weight compounds, and seven higher-molecular-weight compounds. VOC metabolic biomarkers, identified in association with individuals with different physiological states were categorized into two major groups, including (1) activity-specific biomarkers, and (2) metabolomic biomarkers. Two types of activity-specific biomarkers, active-field (AF) biomarkers and torpor (T) biomarkers, were identified as those VOCs uniquely found in the volatile emissions from bats recently engaged in physical activity (flying, feeding, grooming, etc.); and bats primarily in an inactive and unconscious (torpid) state, respectively. Metabolomic (quantitative) biomarkers are VOC metabolites produced and released at varying concentration levels from bats in different physiological states. Two types of metabolomic biomarkers detected included healthy (H) biomarkers and conscious activity (CA) biomarkers. H-biomarkers were defined as volatile metabolites released in greater quantities within VOC emissions from healthy bats, whether from field or cave bats. By contrast, Pd-infected (WNS-diseased) cave bats released either no H-biomarker VOCs or significantly lower quantities in their whole-body emissions than healthy tricolored bats. Conscious activity (CA) biomarkers were associated with emissions from bats with indications of consciousness (wakeful activities), resulting from physiologically active states as in active field bats, or in cave bats with episodes of consciousness occurring during periods of arousal between torpid states. Emissions of CA-biomarker VOCs were greatest in healthy field bats that were continuously awake (lacking torpid states). Cave bats in torpid states with only occasional episodes of arousal exhibited significantly lower levels of these VOCs.

Electronic aroma detection (EAD) data using three-dimensional principal component analysis (PCA) provided strong evidence that healthy, WNS-diseased (Pd-infected), active, and hibernating (torpid) bats had significantly different e-nose aroma signatures, indicative of different VOC (physiological) profiles [2]. They concluded that VOC-detection and analyses using a GC/E-nose provided EAD data that were strongly indicative of different physiological states and provided noninvasive alternative means for early assessments of Pd-infections in individual bats.

A study by Shuba et al. [4] analyzed exhaled breath condensate (EBC) samples obtained from calves to assess the presence of bovine respiratory disease (BRD) using an experimental eight-sensor piezoelectric quartz microbalance (PEQM)-type e-nose. Veterinarians took EBC samples from calves using a special device for collecting exhaled breath into sterile test tubes and froze them in liquid nitrogen for delivery to the laboratory. Samples were defrosted for 15–30 min at room temperature just prior to e-nose gas phase analysis. They tested the use of newly developed experimental polycomposite coatings on PEQM sensors, with either selective or universal sorbents, applied to sensors to impart unique sorption features (increasing sensitivity, selectivity and or efficiency) for detecting different chemical classes of VOCs targeted for detection from EBC veterinary samples. An important parameter in the creation of modified sensors based on piezoelectric quartz resonators (PQR) is the mass of the applied sorbent material used to coat the sensor. The ratio of the masses of individual sorbents used in combination to coat a sensor affected sensor sensitivity and specificity. Since each sorbent material is characterized by certain values of molar-specific sensitivity and selectivity, varying the ratios of the sorbents applied to sensors changed the sorption properties of the resulting polycomposite coating and sensor responses based on it. Thus, it was possible to manipulate the output responses of individual sensors to be selective for certain chemical classes of VOCs. With these established principles, polycomposite coatings applied to the electrodes of PQRs were chosen with select sorbents for modulating sensor output data to obtain the maximum information about the qualitative and quantitative VOC composition of the gas phase derived from EBC sample analytes. The researchers were able to create “visual prints” or eight-sensor radial plots, like breathprint radial plots, of sensor output signals from the modified polycomposite-coated PEQM e-nose sensor array to graphically show differences in sensor response patterns for different veterinary sample types and between individual samples, distinguishing between BRD+ (diseased calves) and BRD− (disease-free calves).

Some of the key findings from the Shuba et al. [4] study indicated that comparative analysis could be used on sets of sensors in the sensor array with different experimental polycomposite coating combinations to improve the efficiency and kinetics of VOC vapor sorption by sensors and to develop relevant polycomposite coatings for detecting specific types of VOCs (targeted for detection) in association with a particular disease. Regression equations were developed to predict the molar-specific sensitivity of the microbalance of VOC vapors using a sensor with a polycomposite coating composed of three distinct sorbent types. A correlation was found between the A**_ij__∑_** parameters of sensor groups used with polycomposite coatings and the biochemical parameters of the EBC biosamples associated with various microbes present in tracheal washes. Furthermore, they demonstrated the possibility of replacing an array of PEQM sensors (containing monocoatings) with sensors coated instead with polycomposite coatings for diagnosing an inflammatory disease in respiratory organs of calves by detecting differences in VOC composition of the gas phase derived from samples of exhaled breath condensate.

### 2.2. Human Clinical Pathology

A new approach to the early prediction of changes in disease state associated with inflammatory bowel disease (IBD) was investigated by Bosch et al. [5] in a pilot study to explore the potential of using fecal VOC emission profiles to predict the future outcomes of the disease course in adults, to facilitate a more timely treatment of patients, and to ultimately improve disease prognosis and outcome. The team utilized a commercial gas chromatography-ion mobility spectrometry (GC-IMS) instrument for VOC analysis of fecal headspace volatiles from IBD patients, initially selected from a group of individuals that met the IBD disease-state criteria. Two samples per patient were taken from a bowel movement for separate instrumental analyses (GC-IMS, E-nose) during a sampling time course of one sampling every four months per patient for one year to predict disease states in future fecal samples. Additional supportive samples were taken from 182 individuals for confirmation of the results following the original time-course sampling. Fecal calprotectin (FCP) levels in fecal samples collected at the first time point displayed different VOC profiles in patients preceding remission compared with those whose IBD disease status remained active.

Data were analyzed using a 10-fold cross validation, whereby nine groups were used for training and the 10th group was used as a test set; the process was repeated 10 times until every group was used as a test set [5]. Discriminatory information from 100 features (parameters) was identified using a Wilcoxon rank-sum test to provide sufficient information content for most applications. The location of these features was exported and plotted onto the original instrument output file. The features were used to train two separate predictive models, based on support vector machine and random forest classifications, respectively. Statistical values such as sensitivity and specificity were calculated from the resulting test probabilities. The observed alterations in fecal VOC profiles of adult IBD patients preceding a change in IBD biochemical disease activity, particularly indicated by levels of the fecal calprotectin (FCP) parameter, were found to be most significant among all of the features identified.

They concluded that alterations in fecal VOC profiles, preceding changes in FCP levels, may be useful to detect disease course alterations at early stages, leading to potential earlier treatment with decreased complications, and reductions in required surgery and hospital admissions [5]. The early prediction of changes in IBD disease states adds to timely treatment adjustment which could potentially improve disease outcome in both adults and children and prevent drug-related side effects. They further proposed that the ability to predict the IBD disease course based on fecal VOC profiles may be partly explained by changes in gut microbiota composition (microbial dysbiosis) due to IBD disease development effects on this major biotic source of fecal VOC emissions.

E-nose sensor temporal drift is a well-known problem and disadvantage of electronic-nose (e-nose) technologies that may affect the accuracy of diagnostic algorithms. A correction for this problem is not always routinely performed in clinical practice. In a related IBD study, Bosch et al. [6] investigated the influence of e-nose sensor drift on the development and accuracy of an IBD disease-specific algorithm in a real-life multi-center cohort of IBD patients. In this cohort, patients undergoing colonoscopy collected a fecal sample themselves prior to bowel lavage. Fecal e-nose profiles were measured using a Cyranose 320 portable e-nose, containing a CBPC 32-sensor array, based on samples from 63 IBD patients and 63 controls measured over four subsequent days. Temporal sensory data were associated with date of measurement, disease state and activity, disease localization, and the diet of the participants. No significant differences were observed between the fecal VOC profiles of IBD patients and those of controls prior to data correction. However, the fecal VOC profiles differed significantly between IBD patients and controls based on six sensors when using corrected sensor-output data. They determined that sensor drift of the C-320 e-nose could be effectively controlled by using regression models to improve data accuracy for differentiating between IBD patients and controls. The residuals of regression models were applied to original patient data to produce data-corrected measurements used for subsequent data analyses. The differences in sensor outputs between sample groups were calculated and logistic regression analyses were performed on six individual sensors that subsequently differed significantly between the treatment groups.

Corrections for e-nose sensor drift significantly improved the accuracy in differentiating between IBD patients and controls based on output differences of six of the 32 sensors (contributing most to discriminations) with an accuracy of 0.68 based on logistic regression [6]. Short-term sensor drift affected fecal e-nose profiles more profoundly than clinical features. They concluded that their results emphasize the importance of making daily sensor drift corrections to improve reliability and repeatability of instrument outputs, both within and across different e-nose studies.

One of the biggest medical problems associated with prescribing appropriate antibiotics to children with bacterial infections is the inability to quickly establish the etiology of the disease necessary for determining appropriate therapeutic treatments. Diagnostic information relating to disease etiology can often be quickly obtained by detecting the unique volatile metabolome of clinical microbial samples using e-nose sensor arrays. Many volatile substances known to be microbial metabolites associated with disease processes may be found in human secretions, including urine samples. This approach was used by Kuchmenko et al. [7] to assess the possibility of using an e-nose device with an array of chemical sensors with certain VOC-sensitive coatings to detect bacterial infections in children based on volatile microbial metabolites. They tested the efficacy of an experimental e-nose (MAG-8) device with seven piezoelectric sensors to detect certain types of bacterial infections in children (ages 1–16) in a hospital, associated with urinary tract and soft tissue infections, by analyzing the equilibrium gas phase (EGP) of urine samples. The experimental device contained a sensor array composed of PEQM sensors coated with different sorbent films (in a numbered sequence) including polyethylene glycol sebacate (PEGSb), triton X-100 (TX-100), dicyclohexane-18-crown-6 (18C6), polyoxyethylene sorbitan monooleate (Tween), methyl red (MR), bromocresol blue (BCB), and multiwalled carbon nanotubes (MCNT). Piezoelectric quartz resonators with an initial oscillation frequency of 10.0 MHz were used. The choice of sensor coatings for the array were based on their high sensitivity to specific chemical classes of VOCs, including volatile biomarkers of diseases in the urine. Sensor films of 18C6 chosen for detection of specific VOC chemical classes included the following: (1) Tween for carboxylic and hydroxy acids detection; (2) MCNT, BCB, and MR for ammonia and amines detection; (3) PEGSb for acids, alcohols, and ketones detection; and (4) TX-100 for detection of nitrogen- and sulfur-containing compounds. The sensor array was trained for detecting certain VOCs, including ethanol, butanol-1, acetone, acetic acid, butyric acid, valeric acid, isovaleric acid, ammonia, diethylamine, piperidine, hydrogen sulfide, phenol, ethyl acetate, dimethylacetal dimethylformamide. After surgical removal of the microbial source of inflammation, microbiological studies were performed to determine the presence and identity of the pathogen associated with each infection.

Volatile biomarkers were identified in the EGP of urine samples, according to identification parameters derived from sensor outputs from the sensor array, using four equations [7]. They found that certain volatile products in urine such as ethanol, butanol, and oxidation products of these VOCs (acetic and butyric acids) were present in almost all samples. The presence of volatile metabolites in the EGP of urine samples was determined both through the processes occurring in the body and the peculiarities of drug metabolism, depending on the drugs used under standard treatment protocols. Hydrogen sulfide in the EGP of the urine was indicative of physical injuries and inflammatory processes. Hydrogen sulfide was also identified in urine samples from patients who had an aseptic inflammatory process without the presence of pathogenic microorganisms. Acetone, ethyl acetate, and isovaleric acid were typical for patients with neurosurgical effects and purulent infections, characterized by tissue inflammation due to pyogenic bacteria, most commonly involving *Streptococcus*, *Staphylococcus*, or more rarely *Pseudomonas* species and pathogenic *E*. *coli*. Volatile metabolites associated with inflammatory processes of bacterial origin were like those associated with temporary metabolic disorders and head injuries. The presence of phenol in the EGP of urine samples was mostly due to neurosurgical pathologies. The occurrence of aliphatic amines and ammonia was indicative of purulent infections and thermal injuries often associated with a massive lesion. The presence of cyclic amines and acetals was difficult to differentiate because they are also the products of the metabolism of drugs used for treatment.

Regression models were built to predict the presence of bacterial infection in children with an error rate of ≤15% with high sensitivity [7]. The resulting models were used to analyze urine samples as a screening tool. The use of a calculated indicator of infection (InfI), based on the results of comparing its values and clinical indicators, yielded a resulting sensitivity of the prediction of 96% and specificity of 50%.

Cancers rank as leading causes of death worldwide and are major public health problems for which diagnoses are challenging, often requiring invasive and expensive tests. The development of noninvasive, early disease detection methods is viewed as paramount to achieving more cost-effective and efficient approaches in clinical diagnostic procedures that minimize pain to patients and greatly improve prognoses through early treatments. Anzivino et al. [8] evaluated the portable CBPC polymer-based Cyranose 320 e-nose for efficacy in distinguishing head and neck cancer (HNC) patients from those with allergic rhinitis and healthy controls. Exhaled breath from all participants was analyzed with the 32-sensor array and plotted with PCA to show that patients diagnosed with head and neck cancer clustered distinctly from the controls and allergic rhinitis. The three patient groups were further discriminated using canonical discriminant analysis (CDA) with a cross-validated accuracy of 75.1% (*p* < 0.01). The area under the curve (AUC) value of the receiver operating characteristic (ROC) curve for discrimination between HNC patients and the other groups was 0.87. Comparisons between patient groups indicated that the distinction between the exhaled VOC spectrum of HNC patients and allergic rhinitis subjects was less sharp than in HNC vs. healthy controls. This was explained by the underlying inflammation of the nasal mucosa that may generate VOCs resulting from oxidative stress. Similar VOCs may also be produced through certain neoplastic processes that increase production of reactive oxygen species and enhance alkane metabolism via cytochrome P450. They concluded that the C-320 e-nose technology demonstrated potential as an effective application for diagnosis of HNC because of the ease of use, quick diagnostic results, noninvasive nature of breath analysis, and the low cost of this tool for clinical operations.

Tyagi et al. [9] conducted a similar efficacy investigation using the portable 10-MOS sensor PEN3 commercially available e-nose operating at 250–550 °C, coupled with Gas chromatography–time of flight-mass spectrometry (GC-TOF-MS), for analysis of gases and VOCs profiles of urinary metabolome for diagnosis of colorectal cancer (CRC). Besides human breath, analysis of urine volatiles is commonly used for disease detection because urine samples are readily obtained noninvasively from patients. They compared different CRC stages with non-cancers using the PEN3 and GC-TOF-MS. The study utilized 96 urine samples acquired at University Hospital Coventry and Warwickshire NHS Trust for analysis with both instruments. They determined that 58 of these were CRC samples and 38 were non-cancerous samples. The CRC samples were further determined to be distributed into 24 early-stage CRC and 34 late-stage CRC samples based on TNM (tumor/node/metastasis) staging. The PEN3 e-nose separated CRC from the non-cancerous group with an AUC value of 0.81. Data from the GC-TOF-MS analyses provided a VOC profile for CRC, from which 23 potential VOCs biomarkers for CRC were identified. Both the PEN3 e-nose and GC-TOF-MS were found to successfully distinguish between the CRC group and the non-cancer group. The results obtained from the PEN3 e-nose and GC-TOF-MS demonstrated high diagnostic accuracy for the separation of CRC and non-cancer patients.

The recent occurrence of coronavirus disease 2019 (COVID-19) that continues as a worldwide pandemic (2019–2023), also informally referred to as Wuhan flu or Chinese flu, caused by the severe acute respiratory syndrome coronavirus (SARS-CoV-2) that originated in Wuhan China, remains a challenge to healthcare providers, with the difficult tasks of (1) detecting this viral pathogen in presymptomatic individuals, symptomless carriers, and early symptomatic patients within worldwide human populations, and (2) applying effective therapeutic treatments at appropriate times based on accurate COVID-19 disease-detection data. Conventional early methods developed for large-scale, mass-screening efforts initially utilized semi-invasive and often painful deep nasal swab sampling methods for diagnostic testing. Nasal sampling was coupled with various PCR-based technologies that generally provided slow delivery of results up to 2 days after sampling, or more rapid antigenic tests such as enzyme-linked immunosorbent assays (ELISA) that provided results within 10 to 30 min, but also required semi-invasive nasal swab sampling and sometimes yielded false-negative test results. The delay in receiving timely and accurate PCR or ELISA results pointed to the need for new noninvasive detection methods providing accurate results in point-of-care testing (POCT) situations where rapid, high-volume, accurate testing results are required. A considerable amount of new research soon followed with the development of new noninvasive early COVID-19 detection methods using e-nose and related electronic devices with a different detection approach based on changes in composition of VOC emissions in the human breath, resulting from COVID-19 pathogenesis effects that simultaneously disrupt a diverse conglomerate of specific human metabolic pathways in different organ systems.

The comprehensive review by Wilson and Forse [10] summarizes results from a wide range of recent international research studies that tested many types of e-nose devices and methods for their capabilities of COVID-19 detection in various healthcare situations and applications. A comparison of the differences in human symptom combinations vs. organs affected and deaths associated with COVID-19 disease vs. those associated with other prior viral pandemics in human history are elucidated. The review also provides considerable detail about the disparate mechanisms of disease development that occur in different human organs due to SARS-CoV-2 viral infections, replications, transmissions, and pathophysiology, and result in the production of COVID-19 disease-specific VOC biomarker metabolites indicative of pathogenesis within the different organ systems affected. They further explain how these COVID-19 specific VOC biomarkers result from the dysregulation (up or down gene regulation) of various human metabolic pathways and serve as effective volatile chemical targets for e-nose detection. The identified connection between VOC biomarker types and metabolic processes, disrupted by COVID-19 disease processes, is based on prior studies identifying known associations between the chemical classes of specific metabolic biomarkers and specific cellular or physiological processes affected. Certain volatile aldehydes, ketones, and sometimes alcohols were consistently identified from multiple studies as key COVID-19-induced metabolites selectively indicative of SARS-CoV-2 infections, particularly when detected simultaneously in the same breath samples of COVID-19+ patients. The available evidence suggested that the VOC chemical classes of identified COVID-19-specific VOC biomarker metabolites were associated and correlated with specific symptom types and individual organs affected by the disease. For example, increased levels of breath aldehydes occur when tissues are damaged by inflammation that affects many organs throughout the body and causes widespread cellular damage and immunosuppression. Increases in breath ketones suggest SARS-CoV-2 viral damage to the pancreas and liver, worsening complications of diabetes and disrupting key metabolic signals causing ketosis, hyperglycemia, or hypoglycemia due to modifying effects on insulin and glucose metabolism. The up-regulation of ethanol is likely associated with COVID-19 effects on acetaldehyde metabolism in the liver, resulting in increases in blood alcohol concentrations.

The Wilson and Forse review [10] also suggested new ways in which e-nose data may be combined with corresponding chemical data (from the same clinical sample) that identifies COVID-19 specific disease biomarkers to increase, confirm, and strengthen the certainty of disease diagnoses. These types of analyses could be simultaneously carried out using dual-technology e-nose devices individually or in combination with instruments with VOC analytical chemistry capabilities. They proposed the additional use of e-nose smellprint signatures to add greater utility in showing differences in individual sensor response patterns (within the sensor array) that demonstrate distinct differences in the composition of VOC mixtures present in clinical breath samples for discriminating between COVID-19+ patients from those lacking SARS-CoV-2 infections.

### 2.3. Detecting Human Dietary Effects on VOC Emissions

Human dietary changes and their effects on the VOC composition of intestinal and urinary gases are poorly explored, particularly in relation to human health and disease. Analysis of these gases is also important as potential chemical biomarkers of normal microbiota activity and as indications of dysbiosis frequently associated with disease states. A variety of methodologies have been used to analyze urinary and intestinal gases with diverse results due the lack of standard procedures for routine clinical applications.

The ingestion of dietary gluten (via wheat products) has been associated with increased gastrointestinal symptoms among celiac disease (CD) patients. McFarlane et al. [11] investigated the effects of a gluten-amended diet (3 g of gluten/day) for 14 days (D0–D14) vs. a control gluten-free diet (GFD) on changes in urinary VOC signatures of CD patients to determine if they were distinguishable from healthy individuals. They used a combination of field asymmetric ion mobility spectrometry (FAIMS), a variant of the ion mobility spectrometry (IMS) technique, deployed as a point-of-care test (POCT) along with GC-TOF-MS to identify specific biomarkers resulting from the gluten challenge (GCh). CD patients were distinguishable from healthy individuals via urinary VOCs analysis. FAIMS revealed significant VOC differences for all time points compared to D0 in GCh patients, whereas GC-TOF-MS revealed significant changes only at D7 and D14. FAIMS showed significant differences in urinary VOCs only at D7 for the control samples, but GC-TOF-MS detected no significant differences for all time points. Twelve VOCs were identified by GC-MS-TOF with significantly altered levels at D7 vs. D0 for GCh patients. These metabolomic alterations persisted for six VOCs until D14, and one (N-methyltaurine) remained altered after D14. The changes in urinary VOC signatures of celiac disease (CD) patients reverted to pre-challenge signatures after the cessation of the GCh challenge. The conclusion was that both FAIMS and GC-TOF-MS methods were capable of detecting changes in VOC signatures in CD patients while undergoing a minimal GCh. These results suggested that urinary VOCs could have a potential application role for monitoring gluten-free dietary compliance in CD patients and detecting recent gluten ingestion. The FAIMS technology has some notable advantages as it is cheaper than GC-TOF-MS, it may be used away from a laboratory setting, and requires no special gases to operate. This makes FAIMS a potentially more useful approach for POCT in the clinical setting. The underlying reasons to explain why the ingestion of gluten may alter urinary VOCs signatures in CD patients was uncertain, but it was postulated that a non-specific, perhaps indirect effect of gluten consumption, such as alterations in the GI microbiome or small bowel duodenal inflammation caused by gluten, may explain the results.

A nutritional intervention, reduced crossover study of healthy subjects by Freire et al. [12] examined the effects of two diets with or without flatulence-inducing legumes, consisting of white beans (250 g), on the production of intestinal gases (flatus). The low-flatulence diet consisted of one portion of pasta, rice, or a white bread sandwich for lunch and soup or lettuce for dinner. A procedure was devised for the direct collection of rectal gases which prevented atmospheric contamination and enabled the simultaneous quantification of five major gases, including oxygen (O_2_), nitrogen (N_2_), carbon dioxide (CO_2_), methane (CH_4_), and hydrogen (H_2_), using direct sampling of flatus collection in Tedlar bags and analysis via gas chromatography–thermal conductivity detection (GC–TCD) and multivariate data analysis. Five healthy subjects were subjected to the two different diets (highly flatulogenic diet and lowly flatulogenic diet) at least one week apart and the corresponding gas intestinal samples were collected. The production of methane and carbon dioxide gases was significantly higher in subjects with the highly flatulogenic diet relative to those with the lowly flatulogenic diet. No significant differences in hydrogen and oxygen production were found between subjects with highly vs. lowly flatulogenic diets. By contrast, nitrogen production was generally lower in subjects under the highly flatulogenic diet. Hydrogen production was correlated with methane production. However, nitrogen production was anticorrelated with concentrations of oxygen and carbon dioxide.

The GC-TCD analysis of intestinal gases relating to diet provided proof-of-concept evidence that this methodology for sampling and measuring flatus major gas components is efficacious [12]. Information relating to intestinal gas composition is useful for understanding the interaction between food intake and intestinal gas production, the gut microbiome, and overall patient health status, and may help in identifying biomarkers of intestinal diseases or other medical conditions such as carbohydrate maldigestion syndrome or bacterial overgrowth in the small intestine. Studies of intestinal gas profiles have traditionally received little attention, but may prove useful in routine clinical tests, including hydrogen- and methane-based testing for detecting gut microbial dysbiosis associated with the development of diseases occurring throughout the body.

## 3. Conclusions

The publications included in this Special Issue provide new information and insights into applications of portable electronic-noses and related VOC detection devices to improve the diagnostic methods for a noninvasive early detection and diagnosis of diseases of wildlife, domesticated animals, and humans prior to symptom development. The use of these various electronic devices for monitoring VOC emissions provides a means for preemptive applications of therapeutic treatments to manage diseases before significant damage has occurred (due to disease) within the affected hosts and to improve the prognoses and recovery from diseased states. The increase in the effectiveness of therapeutic treatments due to application of early disease detection technologies aids in the progress towards the goals of precision medicine to minimize healthcare costs, reducing the duration of hospital visits, and taking advantage of preemptive, prophylactic treatments (prior to the occurrence of disease symptoms) to shorten the disease cycle, reduce secondary infections, and potentially impact disease epidemiology through reductions in exposure rates. Precluding or reducing the times during which presymptomatic individuals are infectious via early treatments may potentially help minimize the exposure of healthy individuals to person-to-person disease transmission.

The increased effectiveness in the detectability of disease-associated VOC-metabolites via the application of electronic devices provides significant improvements as diagnostic tools for early disease detection by accelerating and confirming clinical diagnostic assessments, facilitating the prescription of appropriate treatments, and ultimately increasing the efficiency of healthcare service providers by reducing costs and minimizing patient recovery times.
